# Imaging-Based Diagnosis of a Ruptured Isolated Dissecting Abdominal Aortic Aneurysm: A Case Report

**DOI:** 10.3390/reports9010035

**Published:** 2026-01-24

**Authors:** Marija Varnicic Lojanica, Nikola Milic, Sretina Jovanovic, Milica Ivanovic, Stefan Ivanovic

**Affiliations:** 1 Užice General Hospital, 31000 Uzice, Serbia; 2 Obstetrics and Gynecology Clinic “Narodni Front”, 11000 Belgrade, Serbia

**Keywords:** dissection, abdominal aorta, rupture, ultrasonography

## Abstract

**Background and Clinical Significance:** Acute aortic dissection is the most common and most severe manifestation of acute aortic syndrome. An isolated dissecting aneurysm of the abdominal aorta is defined as a dissecting aneurysm distal to the diaphragm and is an extremely rare disease. Detection of an intimal flap between two lumens using different imaging methods is a definitive diagnostic sign of aortic dissection. A number of studies have validated ultrasound, including point-of-care ultrasound, as the standard initial imaging modality for the diagnosis of aortic dissection. **Case Presentation:** We present a 39-year-old patient who was sent to our institution under the suspicion of renal colic. The clinical findings revealed pale discoloration of the skin with sweating and abdominal pain. An emergency ultrasound showed an abdominal aortic aneurysm with an intimal flap, as well as free perirenal fluid on the left side. Multislice computed tomography aortography was then performed and the findings indicated rupture of a dissecting aneurysm of the abdominal aorta with a large retroperitoneal hematoma. The patient was then sent to a tertiary institution where he underwent emergency surgery and successfully recovered. **Conclusions:** Isolated abdominal aortic dissection is a rare condition with often non-specific clinical presentation, making imaging crucial for diagnosis. Ultrasound plays an important role as an initial imaging modality, as the detection of direct or indirect signs of dissection enables timely referral for CT aortography, confirmation of the diagnosis, and initiation of appropriate treatment.

## 1. Introduction and Clinical Significance

Acute aortic dissection (AAD) is the most common and severe manifestation of the acute aortic syndrome (which also includes intramural hematoma (IMH), penetrating aortic ulcer (PAU), and ruptured thoracic aortic aneurysm) [1]. Aortic dissection (AD) is rarely limited to the abdominal aorta [2]. Isolated dissecting abdominal aortic aneurysm (IAAD) is defined as AAD distal to the diaphragm and is an extremely rare disease [3]. IAAD can be etiologically classified as iatrogenic, traumatic, or spontaneous [4]. The literature suggests that IAAD is most often spontaneous and the most significant risk factors include arterial hypertension (HTN), hyperlipidemia (HLP), and smoking [3]. The clinical presentation of IAAD is highly variable and includes asymptomatic patients with incidental findings of aortic dissection, as well as patients with acute abdominal and back pain, intestinal ischemia, acute renal failure, limb ischemia, and even paraplegia [3,4]. A general physical examination is not sufficient to make a diagnosis of AD. Laboratory tests do not play a significant role in the diagnosis of AD because sensitive and specific laboratory tests for this indication are not yet available. Detection of an intimal flap between two lumens using various imaging modalities (echocardiography, computed tomography (CT), magnetic resonance imaging (MRI), and aortography) is a definitive diagnostic sign of AD. CT is a highly sensitive and specific imaging modality that is most commonly used to evaluate patients with suspected AD [5]. Point-of-care ultrasonography (POCUS) is important for the rapid diagnosis of AD, and the main ultrasound finding in any window is the presence of an intimal flap. Numerous studies have validated ultrasound, including POCUS, as the standard initial imaging modality in the diagnosis of AD [6,7]. Current treatment modalities for IAAD include conservative management with observation, open surgical treatment, and endovascular repair [3]. The aim of this case report is to present a 39-year-old patient with a spontaneous rupture of an isolated dissecting abdominal aortic aneurysm, emphasizing on the importance of imaging modalities in the diagnosis of this disease (especially ultrasonography as the initial imaging modality in this indication).

## 2. Case Presentation

**Figure 1:**
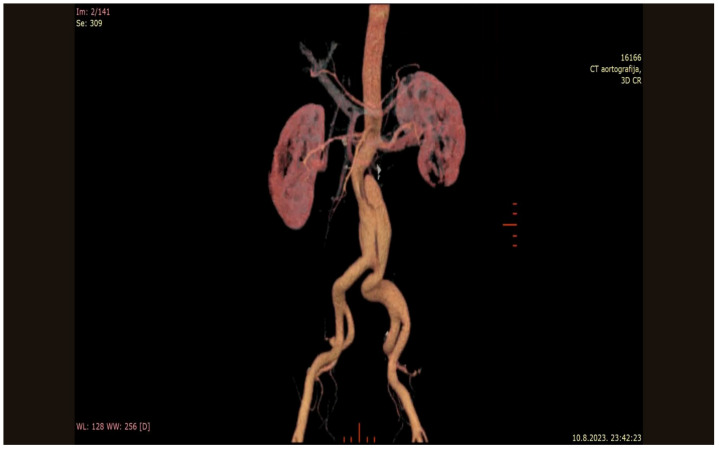
Three-dimensional reconstruction showing the isolated dissecting abdominal aneurism.

**Figure 2:**
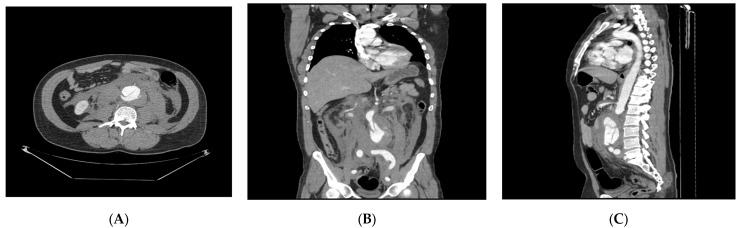
Axial (**A**), coronal (**B**), and sagittal (**C**) scans demonstrating an isolated dissecting abdominal aneurism (located 18 mm distal to the renal arteries origin; aorta measures 58 mm in diameter over a segment approximately 10 cm in length) with the clear visualization of intimal flap, aneurism rupture with active contrast extravasation, and large surrounding retroperitoneal hematoma.

**Figure 3:**
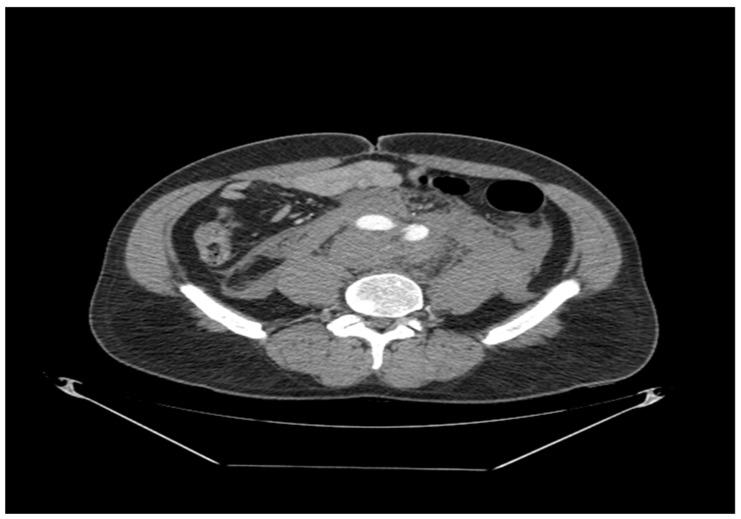
Axial CT scan demonstrating propagation of aortic dissection in left common iliac artery (diameter 28 mm).

A 39-year-old patient was examined in the Emergency Department of our institution due to pain in the lower abdomen, which was more pronounced on the left side and occurred on two occasions on the same day (the first time it occurred in the morning, then it spontaneously passed, and later became unbearable during the afternoon according to the patient, when he reported to the Emergency Medical Service (EMS)). The EMS doctor then referred the patient to our institution under the suspicion of the presence of renal colic. Regarding personal history, smoking, HTN, and HLP were mentioned and previous surgeries were denied. On examination at our institution, the patient was conscious and oriented, with pale skin, diaphoretic, in pain, anxious, and was mildly tachypnoic. On admission, he was hypertensive (blood pressure 170/100 mmHg), with a heart rate of 92 beats/min, respiratory rate of 18 breaths/min, and peripheral oxygen saturation (SpO_2_) of 98% on room air. Initial laboratory investigations revealed leukocytosis with marked neutrophilia, elevated inflammatory markers, increased D-dimer levels, and reduced red blood cell count, hemoglobin, and hematocrit values, consistent with blood loss. Detailed laboratory results are provided in Table S1. Then, native radiography of the urinary tract and emergency ultrasonography (US) of the abdomen were performed. Abdominal US findings were as follows: free perirenal fluid was present on the left side and the abdominal aorta was aneurysmally dilated with a lumen width of up to 44 mm and with a suspected intimal flap; findings on the remaining organs of the abdomen showed no significant pathological changes. Then, an urgent Multi-Detector Computed Tomography (MDCT) aortography was indicated and the findings were as follows: The thoracic aorta had a regular lumen width throughout its course without signs of dissection and contrast medium extravasation; the abdominal aorta was tortuous and sclerotic in the infrarenal segment (about 18 mm from the level of the origin of the renal arteries) and was aneurysmally expanded (lumen width about 58 mm in the segment length of about 10 cm) with signs of dissection (dissecting aneurysm) that propagated into the common left iliac artery (lumen width about 28 mm), and with signs of aneurysm rupture at the level of the left wall with large surrounding retroperitoneal hematoma that propagated to the pelvis (entered the left perirenal space, along both Gerota’s fascia and along both psoas muscles). Two renal arteries were present on the right, with variety and both common iliac arteries were tortuous; findings on the remaining organs of the chest, abdomen, and pelvis were without significant pathological changes (Figure 1, Figure 2 and Figure 3). A surgeon and anesthesiologist at our institution were consulted and the patient was referred to a tertiary institution for emergency surgical treatment. The patient underwent emergency surgery upon arrival at the tertiary institution. A partial resection of the aneurysmally altered infrarenal abdominal aorta was performed, with restoration of blood flow by implantation of a bifurcated Dacron graft in the form of an aortobiiliac bypass, with end-to-end anastomoses to the common iliac arteries. Hemostasis was achieved, the retroperitoneum and abdominal walls were closed in layers, and the patient tolerated the procedure well. The patient was discharged in good general condition and continued regular follow-up according to standard vascular surgery protocols.

## 3. Discussion

Aortic dissection most often originates from the thoracic aorta with consequent distal propagation and frequent involvement of the abdominal aorta. It is classified according to the DeBakey and Stanford classification systems [8]. IAAD is defined as AD distal to the diaphragm and is an extremely rare disease with an incidence of 1.1–4% of all ADs. Also, IAAD does not fit into any of the recognized AD classification systems [3]. IAAD is etiologically classified as iatrogenic, traumatic, or spontaneous [4]. IAADs are most often spontaneous (77–89%), while they are less often traumatic (~17%) or iatrogenic (6–11%), and are most often localized in the infrarenal segment of the abdominal aorta. Spontaneous IAADs are more often associated with HTN and aortic aneurysmal dilatation compared to other types of AD (abdominal aortic aneurysm (AAA) is present in 42% of patients before the onset of IAAD) [4,9]. The most common clinical symptom of IAAD is abdominal pain (50.8%), followed by back pain (30.5%) and chest pain (21.7%), while 41% of patients are asymptomatic. About 71% of patients have negative physical examination findings; the absence of pulse (15.9%), abdominal pulsations (3.1%), and abdominal pain sensitivity (7.9%) are rarely present [8]. The incidence of aortic, iliac, and femoral ruptures of infrarenal IAADs is 17%, with a mortality rate of 44%. The literature also provides evidence of a correlation between the simultaneous presence of AAA and infrarenal IAAD and an increased risk of aortic rupture [10]. The most significant risk factors for the occurrence of IAAD are smoking, HTN, and HLP and are considered to contribute to the development of atherosclerosis and spontaneous AD [3]. The literature reports that the median age of patients with infrarenal IAAD is similar to the median age of patients with DeBakey type III AD, which is 58 years old [10]. Also, the literature reports that the development of atherosclerotic changes in the aorta is significantly correlated with age (the incidence of pronounced atherosclerotic changes in the abdominal aorta in patients younger than 55 years is about 27%) and that advanced atherosclerotic changes occur 2–3 times more often in heavy smokers compared to non-smokers (especially in the abdominal aorta) [11]. Shaiea et al. reported that although abdominal aortic aneurysms and dissections most commonly occur in older patients, isolated abdominal aortic dissection may also present in younger individuals, particularly in the presence of risk factors such as arterial hypertension, hyperlipidemia, and smoking [12]. The aforementioned may indicate a correlation between the presence of risk factors (smoking, HTN, and HLP) and a positive family history of aortic diseases and the described findings on MDCT aortography (pronounced atherosclerotic changes, tortuosity, AAA, and IAAD) in our patient, which were unusual considering his age (39 years). The detection of an intimal flap between two lumens using different imaging methods (echocardiography, CT, MRI, and aortography) is a definitive diagnostic sign of AD [5]. CT aortography is the imaging method of choice in the diagnosis of AD, with a sensitivity and specificity of 100% and 98–99%, and is optimally performed by administration of intravenous (IV) contrast medium, multiphasic image acquisition, and using “ECG gating”. The dynamic nature of the aorta, especially the ascending aorta and the aortic arch, affects the appearance of movement artifacts (which can be reduced by applying “ECG gating”) that can be misinterpreted as dissection (7–12% of patients are misdiagnosed due to suboptimal acquisition and analysis of CT images) [13]. Adequate acquisition and analysis of CT images allow for clear differentiation of AD, IMH, and PAU [14,15]. The main features of AD are the presence of an intimal rupture that leads to damage to the media with the formation of an intimal flap and blood flow through the true and false lumen. The true lumen can be distinguished on the basis of size (the true lumen is usually smaller in diameter than the false one); it is in direct communication with the lumen of the aorta and the movement of the intima towards its lumen is observed. The false lumen is usually larger in diameter compared to the true lumen; a “cobweb sign” is usually present, it demonstrates a more pronounced convexity of the surface of the intimal flap towards its lumen and there is an impression of twisting around the true lumen. Hypodensity of the true lumen is also observed during peak aortic enhancement due to slower indirect flow. IMH is characterized by the presence of a growing hematoma within the aortic wall, which is best seen on native CT images, and is without clear visualization of direct communication between the false and true lumen on enhanced CT images. PAU is a lesion caused by atherosclerosis that penetrates through the internal elastic lamina of the aortic wall and is often in the vicinity of the calcified plaque. The occurrence of IMH in adjacent segments of the aorta is also possible [10]. The finding of a ruptured AAA is obvious and includes massive periaortic bleeding, including the pararenal and perirenal spaces [16]. MRI is the most sensitive imaging method in the detection of AD (sensitivity 100% and specificity 94%), but it is rarely used as an initial imaging method because it cannot be used in hemodynamically unstable patients and in patients with metal implants or prostheses. It is limited in availability; it requires a longer time of processing the images and there are often problems with patient monitoring during acquisition [5]. POCUS is important for the rapid diagnosis of AD and the main US finding in any window is the presence of an intimal flap that represents a mobile linear structure independent of the aortic wall. Cardiac complications of AD, especially in AD Stanford type A, such as aortic regurgitation, pericardial effusion, and myocardial ischemia, should also be detected with POCUS [6]. Transesophageal US (TEE) is a highly sensitive method (>95%) in the detection of AD Stanford type A. Transthoracic US (TTE) in the detection of AD Stanford type A has a sensitivity of 52–90% and a specificity of 63–100%, and in the detection of AD Stanford type B, data is limited with a sensitivity of 33–70% is reported [7]. A number of studies have validated US, including POCUS, as the standard initial imaging method in the diagnosis of AD. The literature reports that the use of POCUS reduces the time to diagnose AD by about 143 min, which is significant considering the 1–2% increase in mortality rate per minute from the onset of symptoms in these patients. It is also reported in the literature that the presence of indirect US signs indicates high-risk patients who require urgent application of other imaging methods, surgery, and other medical interventions. Also, high-risk patients without US findings for AD require urgent further diagnostic work-up and POCUS findings cannot rule out AD [7]. Alasasfeh et al. reported the case of ruptured aneurysm that was diagnosed after the onset of hemodynamic collapse, resulting in a fatal outcome, with delayed diagnosis and limited availability of diagnostic and surgical resources highlighted as key contributors to the unfavorable outcome [17]. In the case of our patient, the abdominal US findings (presence of an intimal flap, AAA, and the presence of free fluid on the left side of the perirenal space) indicated the existence of AD and raised the suspicion of the existence of an AD rupture, which enabled timely referral of the patient to MDCT aortography and subsequent diagnosis of IAAD rupture, which highlights the importance of US as an initial imaging method in the diagnosis of AD. Moreover, the contemporary literature confirms the high diagnostic value of POCUS in emergency settings and its role in the prompt identification of abdominal aortic aneurysms, as well as in guiding subsequent diagnostic and therapeutic management [18].

## 4. Conclusions

IAAD is an extremely rare disease with often absent or non-specific clinical signs and symptoms, which highlights the importance of imaging methods in the diagnosis of this disease. The presence of direct or indirect US signs of AD can raise the suspicion of the existence of AD and enable timely referral of the patient to other imaging methods that will confirm the diagnosis of AD (such as CT aortography) and timely treatment of this disease, which emphasizes the importance of US as an initial imaging method in this indication. Early use of ultrasound, including POCUS, enables recognition of direct and indirect signs of aortic pathology and facilitates timely referral for definitive diagnostic evaluation and emergency treatment.

## Data Availability

Data presented in this study are available from the corresponding author upon request due to privacy concerns.

## Supplementary Materials

The following supporting information can be downloaded at: https://www.mdpi.com/article/10.3390/reports9010035/s1. Table S1. Laboratory analyses included complete blood count, biochemical analyses, and coagulation status

## Abbreviations

The following abbreviations are used in this manuscript:

AAD	Acute aortic dissection
AD	Aortic dissection
IAAD	Isolated dissecting abdominal aortic aneurysm
AAA	Abdominal aortic aneurysm
IMH	Intramural hematoma
PAU	Penetrating aortic ulcer
HTN	Hypertension
HLP	Hyperlipidemia
CT	Computed tomography
MDCT	Multi-detector computed tomography
MRI	Magnetic resonance imaging
US	Ultrasonography
POCUS	Point-of-care ultrasonography
EMS	Emergency Medical Service
CRP	C-reactive protein
IV	Intravenous
ECG	Electrocardiography
TEE	Transesophageal echocardiography
TTE	Transthoracic echocardiography
