# Supplementary material for: Imaging-Based Diagnosis of a Ruptured Isolated Dissecting Abdominal Aortic Aneurysm: A Case Report
Source: Reports (MDPI). 2026 Jan 24;9(1):35. doi: 10.3390/reports9010035 (PMC12921952; doi:10.3390/reports9010035)
Supplement: Supplementary file 1 [file reports-09-00035-s001.zip › reports-4094835-supplementary.pdf]

Table 1. Laboratory analyses included complete blood count, biochemical analyses, and coagulation status

Parameter	Result	Reference range	Units
WBC	15.4*	3.4–9.7	$\times 10^9$/L
Lymphocytes (%)	3.00*	20.0–46.0	%
Monocytes (%)	3.60	2.0–12.0	%
Neutrophils (%)	93.30*	44.0–72.0	%
Eosinophils (%)	0.00	0.0–7.0	%
Basophils (%)	0.10	0.0–1.0	%
Lymphocytes (#)	0.50*	1.20–3.40	$\times 10^9$/L
Monocytes (#)	0.60	0.10–0.80	$\times 10^9$/L
Neutrophils (#)	14.30*	2.10–6.50	$\times 10^9$/L
Eosinophils (#)	0.00	0.00–0.40	$\times 10^9$/L
Basophils (#)	0.00	0.00–0.10	$\times 10^9$/L
RBC	3.70*	4.34–5.72	$\times 10^{12}$/L
Hemoglobin	118*	138–175	g/L
Hematocrit (HCT)	0.344*	0.415–0.530	L/L
MCV	93.0	83.0–97.2	fL
MCH	31.90	27.40–33.90	pg
MCHC	343.0	320.0–360.0	g/L
RDW	13.20	12.00–15.50	%
Platelets (PLT)	214	158–424	$\times 10^9$/L
MPV	9.60	9.10–12.10	fL
PCT	0.210	0.107–0.441	%
PT	11.5	10.4–13.0	s
INR	1.01	0.80–1.20	—

aPTT	22.5	22.0–32.0	s
Fibrinogen	3.2	2.0–4.0	g/L
D-dimer	1.35*	<0.5	mg/L FEU
Glucose	8.3*	3.9–6.1	mmol/L
Urea	5.3	2.5–7.5	mmol/L
Creatinine	98	59–104	µmol/L
eGFR	>60	—	mL/min/1.73 m^2
Total bilirubin	9.3	0.0–20.5	µmol/L
Direct bilirubin	xxx	0.0–5.0	µmol/L
Total proteins	54*	62–81	g/L
Albumin	38	35–53	g/L
Sodium	139	135–148	mmol/L
Potassium	4.2	3.5–5.1	mmol/L
Chloride	101	98–107	mmol/L
Calcium	2.05*	2.15–2.65	mmol/L
Phosphate	1.36	0.80–1.55	mmol/L
Magnesium	0.70	0.70–1.16	mmol/L
Bicarbonate	22	21–31	mmol/L
AST	15	0–37	U/L
GGT	18	0–55	U/L
LDH	344	220–460	U/L
CK	202*	0–200	U/L
CRP	20.6*	0.0–5.0	mg/L
